# Toward a Generalizable Prediction Model of Molten Salt Mixture Density with Chemistry‐Informed Transfer Learning

**DOI:** 10.1002/cphc.202500273

**Published:** 2025-10-14

**Authors:** Julian Barra, Shayan Shahbazi, Anthony Birri, Rajni Chahal, Ibrahim Isah, Muhammad Nouman Anwar, Tyler Starkus, Prasanna Balaprakash, Stephen Lam

**Affiliations:** ^1^ Department of Chemical Engineering University of Massachusetts Lowell 1 University Ave, SOU‐202E Lowell MA 01854 USA; ^2^ Nuclear Science & Engineering Division Argonne National Laboratory Lemont IL 60439 USA; ^3^ Nuclear Energy and Fuel Cycle Division Oak Ridge National Laboratory Oak Ridge TN 37830 USA; ^4^ Department of Mechanical and Nuclear Engineering Tennessee Tech University Cookeville TN 38505 USA; ^5^ Computing and Computational Sciences Directorate Oak Ridge National Laboratory Oak Ridge TN 37830 USA

**Keywords:** inorganic materials, machine learning, molten salts, nuclear energy, transfer learning

## Abstract

Optimally designing applications of molten salts requires knowledge of their thermophysical properties over a wide range of temperatures and compositions. There exist significant gaps in existing databases and this data can be challenging to experimentally measure due to high temperatures, salt corrosivity, and salt hygroscopicity. Existing databases have been used to create Redlich–Kister (RK) models for mixture density showing improved accuracy with respect to ideal mixing assumptions, but these models require subcomponent data measurements for each new system, therefore lacking generality. In order to address generalizability and data sparsity, a transfer learning procedure is proposed to train deep neural networks (DNNs) using a combination of semi‐empirical relationships (RK), data from the thermophysical arm of the molten salt thermal properties database and universal ab initio properties of component mixtures taken from the joint automated repository for various integrated simulations (JARVIS) classical force‐field inspired descriptors database to predict density in molten salts. Herein, it is shown that DNNs predict molten salt density with an r^2^ over 0.99 and a mean absolute percentage error under 1%, outperforming alternative methods.

## Graphical abstract

Deep neural networks for the prediction of molten salt density are trained through a transfer learning process by first training a model on data generated through Redlich–Kister expansions and then reusing layers of that model to train another model directly on the reference data, achieving improved generalizability.

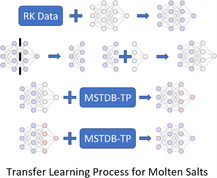

## 1 Introduction

Molten salts are liquid mixtures of cations and anions which have recently commanded widespread interest in their use as energy materials. Owing to their desirable thermophysical properties, such as high heat capacity, and low vapor pressure,^[^ ^1^ ^]^ their potential applications include advanced nuclear reactors, thermal energy storage, pyrochemical reprocessing of used nuclear fuel, carbon capture, and molten salt batteries.^[^ ^2^, ^3^, ^4^, ^5^ ^]^ Their wide applicability across a range of clean energy areas has raised the need for data on their material properties for the purposes of design and optimization. However, obtaining data solely through experiments involves challenging measurements at very high temperatures for salts that are often toxic and very corrosive. Also, given that the experiments involve high costs in both equipment and labor, the massive compositional space of molten salts make their exploration through experimentation alone impractical. These issues have driven the use of alternative methods for modeling and evaluating molten salt properties, including molecular dynamics (MD) for atomistic simulations to evaluate material properties.^[^ ^6^ ^]^ Here, while the MD approach has proven successful,^[^ ^7^, ^8^, ^9^, ^10^, ^11^, ^12^, ^13^, ^14^, ^15^, ^16^ ^]^ it involves a higher computational cost in both its classical and ab‐initio based implementations than what can be afforded with other methods discussed in this work^[^ ^8^ ^]^ and thus, is unfeasible for the prediction of more than a small number of systems and properties. As such, the search for computationally more efficient methods to navigate the temperature and compositional space of molten salts remains critical for the timely development of next‐generation molten salt technologies.

The semi‐empirical Redlich–Kister (RK) method was recently demonstrated for predicting molten salt mixture density $\rho_{\text{micx}}$ and viscosity $\mu_{\text{mix}}$ for up to 3 pseudo‐components using equations fitted to data available in the molten salt thermal properties database‐thermophysical (MSTDB‐TP).^[^ ^17^, ^18^ ^]^ The MSTDB‐TP contains property correlations fitted from over 140 published studies for thermophysical properties of interest in molten salts, which include macroscopic density, viscosity, thermal conductivity, and heat capacity. The database contains properties on over 448 different molten salt systems ranging from pure salts (e.g., LiF), to mixtures of up to four pseudo‐components (e.g., LiF‐NaF‐KF‐UF_4_) with material densities being the most well‐characterized property. This is due to its fundamental importance in relation to thermodynamic properties and relative ease and accuracy of measurement. The empirical equation for macroscopic density as a function of temperature for a given system in MSTDB‐TP is shown below

(1) ρ(T)=A−B×T

where *A* and *B* are fitted coefficients for each composition.^[^ ^15^ ^]^ This equation can be used to generate density data across temperature, for which RK expansions can be used to interpolate between both composition and temperature, calculating mixture density as the sum of the density calculated under ideal mixing assumptions and an excess density representing the deviation from ideality due to pseudo‐component interaction effects (Equation 2). Ideal density is calculated according to Equation (3), and the excess density is calculated according to Equation (4) and (5)

(2) $$\rho_{\text{mix}}=\rho_{\text{id}}+\rho_{\text{ex}}$$

(3) $$\rho_{\text{id}}=(\sum_{i=1}^{S}x_{i}M_{i})/(\sum_{i}^{S}\frac{x_{i}M_{i}}{\rho_{i}})$$

(4) $$\rho_{\text{ex}}=\sum_{a=1}^{S-1}\sum_{b=2}^{S}x_{a}x_{b}\sum_{j=1}^{n}L_{j}^{\text{ab}}{\left(x_{a}-x_{b}\right)}^{j-1}$$

(5) $$L_{j}^{\text{ab}}=A_{j}^{\text{ab}}+B_{j}^{\text{ab}}T$$

where *S* is the total number of components in the system, *x*_i_ and *M*_i_ are the molar concentration and molar mass of component *i* with experimentally measured density $\rho_{i}$, *x*_a_ and *x*_b_ are the molar concentrations of components *a* and *b,* which are considered a pseudobinary subsystem of the system of size *S*, and $L_{j}^{\text{ab}}$ are linear temperature‐dependent interaction parameters associated with pseudobinary subsystem components *a* and *b.* The coefficients in $L_{j}^{\text{ab}}$ are fitted to available experimental data (typically pseudobinary, ternary, or both). As such, RK expansions improve the accuracy of the ideal mixing model (Equation (3)) by accounting for non‐ideal contributions with system‐specific parameters (Equation (4) and (5)). Herein, the RK formalism in Equation ((2), (3), (4))–(5) can be used to 1) interpolate between experimental data points collected for lower order mixtures (typically pseudobinary or pseudo‐ternary) at different temperatures and compositions, or 2) to extrapolate to higher‐order mixture using the coefficients corresponding to the pairings of all compounds present in that mixture (typically fitted from data of pseudo‐binary systems). Thus far, RK expansions have been used to perform both kinds of property predictions, and in both cases, the approach shows higher accuracy than assuming ideal mixing, reflecting the demonstrated non‐ideality in molten salt behavior resulting from interactions between pseudo‐components.^[^ ^19^ ^]^ However, a key limitation of such semi‐empirical approaches is in cases where models have not been developed for lower order (binary or ternary) to inform on higher order mixtures, or when data is sparse, which can lead to significant interpolation error, particularly towards increasingly non‐ideal systems (e.g., LiF‐NaF‐ZrF_4_).^[^ ^20^, ^21^ ^]^

The present work uses density to demonstrate proof of concept for a method for training neural networks for property prediction in molten salts. The method proposed here aims to further improve the accuracy and generalization capability of RK expansions at a similar computational cost by using MSTDB‐TP data to train a deep neural network (DNN) for interpolating properties across a wide range of compositions and temperatures. Li et al define “in‐distribution generalization” as the capacity to predict the correct response on a learned task for novel “test inputs” which are sampled from the same distribution as the training examples, and generalization more broadly as related to this ability to predict well in samples as different from those used to train the model as possible.^[^ ^22^, ^23^ ^]^ DNNs are a type of machine learning model that consists of a neural network composed of multiple hidden layers, with the general consensus being that a network composed of two or more hidden layers can be considered “deep”, although this definition can vary.^[^ ^24^, ^25^ ^]^ This multilayer structure allows for hierarchical feature learning, which some argue is what defines DNNs and deep learning more generally.^[^ ^26^ ^]^ While machine learning (ML) methods have been previously used for predicting the properties of materials,^[^ ^27^, ^28^, ^29^, ^30^, ^31^ ^]^ including ionic liquids,^[^ ^32^, ^33^, ^34^, ^35^ ^]^ compounds, and simple molecules,^[^ ^36^, ^37^ ^]^ the prediction of mixture properties is scarce.^[^ ^38^ ^]^ The limited applications of ML for liquid mixtures are likely owed to challenges in representing multiple components in dynamically disordered systems that lack defined structure in the solution phase. In molten salts, ML has largely been used to learn the potential energy surface with interatomic potentials used in MD simulations, thereby learning the atomic energies as a function of the evolving local chemical environment for a given system.^[^ ^8^, ^9^, ^10^, ^39^ ^]^ Recently, ML prediction of molten salt properties based on simple atomistic descriptors was demonstrated for a simple property (melting point) and a specific class of salts (mixed alkali halide reciprocal salts).^[^ ^40^ ^]^ However, efficiently predicting the properties of all molten salt mixtures directly based on their composition and thermodynamic conditions is challenged by the lack of large datasets that are typically required for training DNNs. To deal with these challenges, this work proposes a methodology for the prediction of molten salt properties that addresses these concerns about generalizability (due to a relatively small training set limited by experimental data), through transfer learning (TL)^[^ ^41^ ^]^ of insights from state‐of‐the‐art semi‐empirical relations (i.e., RK expansion); and by encoding molten salt mixtures using chemical properties of its components as descriptors, therefore in principle allowing for the applicability of the model to any material. The approach for generalizability is based on the theory of molten salt behavior, which proposes a relationship between salt mixture properties and atomistic features of the components within the salt mixture, such as charge (or electronegativity) and ionic radius (or polarizability).^[^ ^40^, ^42^, ^43^ ^]^ A key objective of this workflow is to create models to predict salt mixture properties with a higher accuracy than RK expansions, while retaining the generalizability of ideal or semi‐empirical non‐ideal mixing models, at a similarly low computational cost.

## 2 Results and Discussion

First, a dataset is created using all available compositions from the MSTDB‐TP (Thermophysical) v3.0^19^ for which an empirically fitted equation for density is available. The MSTDB‐TP contains 374 such different molten salt compositions, 26 of which are pure salts, 182 are pseudobinary salts, 160 are pseudoternary salts, and 6 of which are pseudoquaternary salts. The equations are sampled at 25 K intervals across the applicable temperature range (ATR) for each system, starting from the lower bound of the ATR. The temperature and molar fractions of the compounds in the mixture are added as descriptors. To make the inputs for chemical species applicable across the periodic table, chemical information of the salts is encoded using JARVIS Classical Force Field Inspired Descriptors (JARVIS‐CFID)^[^ ^44^ ^]^ database of ab‐initio calculations, which contains 1557 chemical‐structural‐charge descriptors for any compound (e.g., LiF) at 0 K. These descriptors are further down‐selected; details of the down‐selection process are provided in section S1 of the supplementary information, along with the list of the final descriptors in section S2. A DNN model is developed for up to 4 components by concatenating descriptors of each component compound and using zero‐padding to represent lower‐order systems in the input. After down‐selection, a total of 138 descriptors are added from JARVIS‐CFID for each component. Tests of whether the inclusion of these descriptors is effective can be found in section S3 of the supporting information. A test set consisting of 20% of the overall dataset is withheld during training and used to check model performance on unseen data. All chemically equivalent orderings of the input are added to both training and test sets (e.g., for a pseudo binary mixture where **C**_**1**_ and **C**_**2**_ are the set of descriptors for components 1 and 2, respectively, both **[C**_**1**_, **C**_**2**_ **, 0, 0]** and **[C**_**2,**_ **C**_**1**_, **0**, **0]** are included, and so are **[C**_**1,**_ **0,** **C**_**2**_ **, 0]**, **[C**_**2,**_ **0, 0, C**_**1**_ **]**, etc.) to ensure permutational invariance, in a manner similar to previous work in the literature.^[^ ^38^, ^45^ ^]^ This generates a maximum of 24 permutations of each original data point, but several of the permutations of pure and pseudobinary salts are repetitions of other permutations, with those repetitions being discarded so no two data points are exactly the same; therefore, permuting pure salts only four times and pseudobinary salts 12 times. The permutation process is performed after the data has been sampled across temperature and split in training and test sets to prevent permutations of the exact same density data point from appearing in both sets, which would raise the issue of data leakage (the results of attempting a more extreme data leakage prevention strategy by splitting the dataset before both temperature sampling and permutation can be found in section S4 of the supporting information). The final ‘experimental’ dataset contains 52 320 data points.

**Figure 1:**
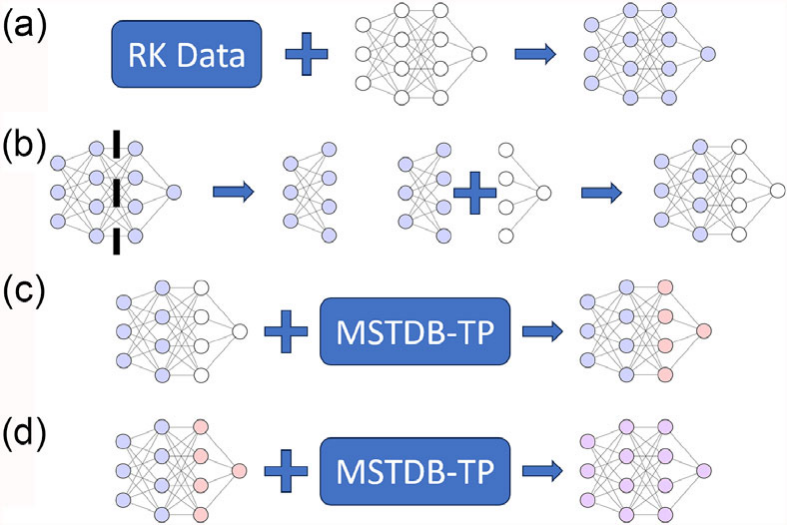
TL workflow performed whereby knowledge from RK models is transferred to the DNN and updated with a smaller experimental dataset. The steps are as follows: a) The DNN is trained on density data generated across temperature using binary RK expansions. b) The latter half and the output neuron of the trained model are discarded, and the first two layers are frozen and used to create a new model by adding untrained layers and an output neuron. c) The new model is trained on the dataset generated with the MSTDB‐TP. d) The model is trained again on the dataset generated with the MSTDB‐TP, this time with all layers unfrozen.

To improve generalization capability, reduce overfitting in data‐sparse regimes, and regularize the DNN model, a TL process based on the description given in Géron^[^ ^46^, ^47^ ^]^ is implemented as shown in **Figure** **1**, in which an approximate DNN is initially trained with large quantities of RK modeled data sampled across temperature and composition. This approximate DNN model is then refined with the smaller experimental dataset sampled from MSTDB‐TP. During TL, a DNN composed of four hidden layers is trained on the RK dataset across 5000 epochs using a learning rate of (*η *= 5E‐4), and the first two hidden layers of the initial DNN are retained and used to create a new network by adding three new layers with an additional output neuron. As shown in Figure 1c,d, this new DNN is then trained on the MSTDB‐TP (comparatively smaller experimental dataset) two times – first with the first two layers frozen to retain the information of the old model and across 10 000 epochs using a learning rate of *η *= 5E‐4, evaluating the validation loss on 20% of the training set at each ten epochs and preserving the model with the lowest value for the validation loss among the ones inspected, and then a second time across 250 epochs with all layers unfrozen using a very small learning rate (*η *= 2E‐6) to fine‐tune it. Throughout all training steps, a batch size of 400 was used, and the mean squared error (MSE) is chosen as the loss function. By preconditioning the model with large quantities of RK data, approximate relationships can be learned between the descriptors and mixture density, and the need for benchmarked experimental data from MSTDB‐TP can be minimized. The RK dataset is generated using the available parameters for binary RK expansions given in the released version of the MSTDB‐TP v.3.0^19^. Density data is created for the pseudo‐ternary systems in the MSTDB‐TP, at increments of 10% molar fraction for each compound, and including all chemically equivalent orderings of components in each data point. The chemical information of the salts is encoded using JARVIS‐CFID descriptors as done for the experimental dataset. The final RK dataset used to train the DNN consists of 134 784 data points.

As such, the DNN trained on the RK dataset is composed of four hidden layers with 128 neurons each, whereas the version trained on the MSTDB‐TP data is composed of five hidden layers (two taken from the initial model plus three new layers). The networks are implemented with L2‐norm regularization as a measure against overfitting. The number of nodes, learning rates, and the activation function used to build the model (“softplus”) were chosen by running the entire process several times to see which combination of hyperparameters led to the least validation loss, evaluated on a validation set consisting of 20% of the training set, chosen at random. A summary of all hyperparameters used for training the models can be found in **Table** **1**.

**Table 1:** List of different hyperparameters and factors used in the building and training of the model presented in this article, reproduced in this table for the purposes of easier reproducibility of our work.

Parameter	Value
Python framework	TensorFlow
Nr. of layers (trained in RK)	4
Final Nr. of layers	5
Nr. of units per layer	128
Epochs (RK training)	5000
Epochs (2nd round of training)	10 000
Epochs (Fine‐tuning)	250
Learning rate (RK training)	5E‐4
Learning rate (2nd round of training)	5E‐4
Learning rate (fine‐tuning)	2E‐6
Batch size	400
L2 norm regularization coefficient	0.01
Test set size	20%
Activation function	Softplus
Optimizer	Adam

**Figure 2:**
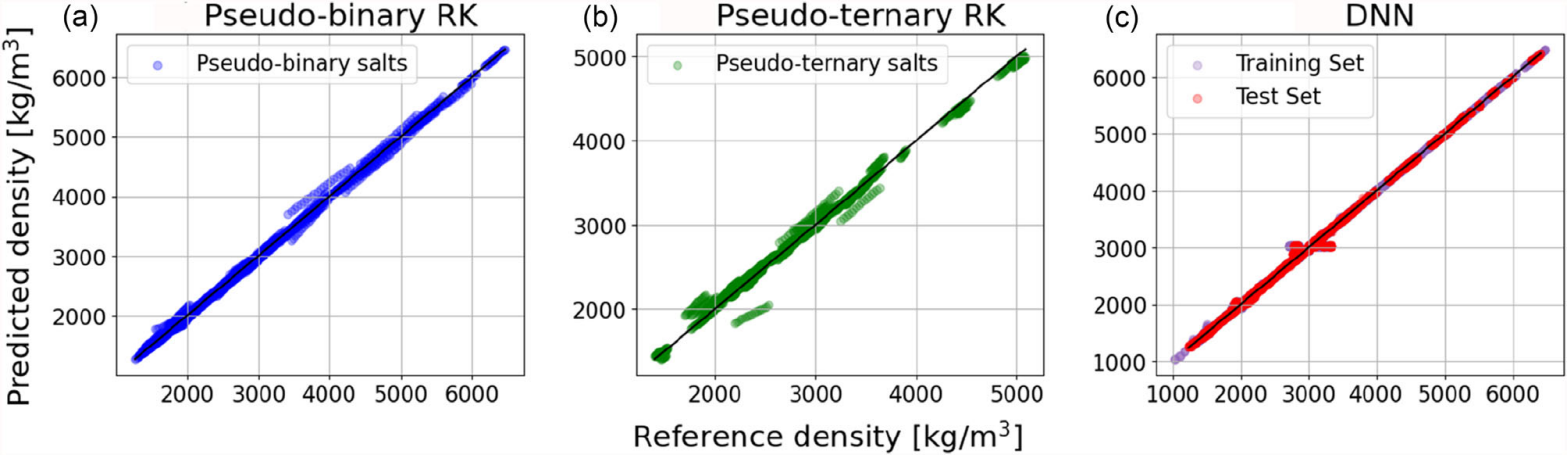
Parity plots (predicted vs. reference values) for density [kg m^−3^] as predicted by a) RK expansions for pseudobinary mixtures using binary interaction coefficients, b) RK expansions for pseudo‐ternary mixtures using ternary interaction coefficients, and c) a DNN across the entire MSTDB‐TP dataset containing both pseudobinary and pseudo‐ternary mixtures.

In **Figure** **2**, the performance of RK is compared to DNN for predicting the density of pseudobinary (Figure 2a), and pseudo‐ternary (Figure 2b) molten salts in the MSTDB‐TP with mean absolute error (MAE), mean absolute percentage error (MAPE), and r^2^ compared in **Table** **2** (comparisons and analysis of ideal mixing can be seen in section S5 of the supporting information). Here, the best available RK model is used for each system. Namely, the pseudo‐ternary systems are fitted with system‐specific pseudo‐ternary interaction parameters for comparison.

**Table 2:** MAE, MAPE, coefficient of determination (r^2^), and maximum errors calculated on the predictions made by a DNN, RK expansions using binary interaction coefficients (binary), and RK expansions using ternary interaction coefficients (ternary).

	Overall		
	DNN	RK Binary	RK Ternary
MAE $\left[\frac{\mathrm{kg}}{m^{3}}\right]$	6.739	20.87	43.10
MAPE [%]	0.265%	0.768%	1.711%
r^2^ [−]	0.9993	0.9987	0.9900
Max Error $\left[\frac{\mathrm{kg}}{m^{3}}\right]$	Training set: 308.6 Test set: 313.9	290.9	488.5

The results show the higher overall accuracy of the DNN, which predicts density with a MAE of 6.739 kg m^−3^ and a MAPE of 0.265%, which is significantly lower than for the predictions made by RK for binary systems (MAE = 20.87 kg m^−3^, MAPE = 0.768%) and for ternary systems (MAE = 43.10 kg m^−3^, MAPE = 1.711%). In ternary systems, RK exhibits higher error due to increased complexity of pair interactions between the mixture components, which is readily overcome by the increased expressiveness afforded by the DNN model. Furthermore, while ternary RK models require significant amounts of data from the system's lower‐order mixtures, the DNN can learn from the entire database and interpolate relationships in other systems containing the same or chemically similar component salts. Furthermore, the DNN achieves an error below experimental uncertainty, which typically ranges from 1% to 2% and can be as high as 6% for the higher‐order mixtures in the MSTDB‐TP at the lowest temperatures.^[^ ^48^ ^]^ Newer methods developed for pycnometric density measurement in molten salts achieve an overall uncertainty of 0.3%,^[^ ^49^ ^]^ which is higher than the error achievable by DNN, but not by RK, for neither binary nor ternary mixtures. The more widely used Archimedean method can achieve uncertainties almost as low as 0.5%, still under RK and Ideal mixing errors, yet over DNN error. The potential for high accuracy through the use of DNNs paves the way for using models trained on molten salt property datasets of diverse compositions through this process and well‐validated to benchmark other modeling methods in the future.

**Figure 3:**
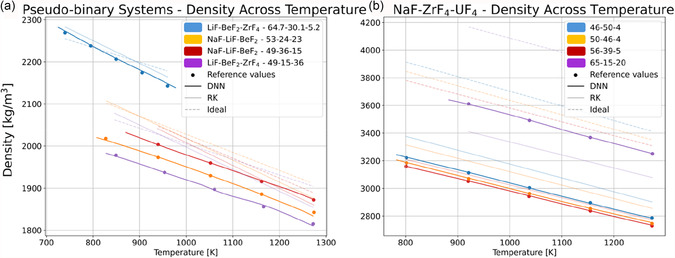
Molten salt density versus temperature for a) LiF‐BeF_2_‐ZrF_4_, NaF‐LiF‐BeF_2_, and LiF‐BeF_2_‐ZrF_4_ compositions using RK expansion with pseudobinary interaction parameters, and b) NaF‐ZrF_4_‐UF_4_ using RK expansion with ternary interaction parameters.

To further assess model performance (smoothness, physical correctness, predictability), the ability of the DNN to capture the dependence of density on temperature and composition is examined in more detail for a variety of prototypical systems. In **Figure** **3**, DNN‐based ρ(T,x) predictions for LiF‐BeF_2_‐ZrF_4_, NaF‐LiF‐BeF_2_, LiF‐BeF_2_‐ZrF_4,_ and NaF‐ZrF_4_‐UF_4_ are shown and compared to the ideal and RK models recently developed in Ref. [20] containing binary interaction parameters (Figure 3a) and ternary interaction parameters (Figure 3b). The MAE and MAPE are calculated for all prediction models across 100 temperature samples within the ATR in the MSTDB‐TP and shown in **Table** **3**.

**Table 3:** MAE and MAPE calculated for the predictions made by the DNN, the binary RK expansion and ideal mixing (ideal) on the systems shown in Figure 3.

	Overall		
	DNN	RK	Ideal
MAE $\left[\frac{\mathrm{kg}}{m^{3}}\right]$	2.275	80.50	334.2
MAPE [%]	0.093%	2.938%	9.708%

Here, DNN exhibits the lowest error with MAE = 2.275 kg m^−3^ and MAPE = 0.093% compared to RK models, which have MAE = 80.50 kg m^−3^ and MAPE = 2.938%, and ideal mixing, which has MAE = 334.2 kg m^−3^ and MAPE = 9.708%. As shown in Figure 3, the DNN more accurately captures both the temperature and composition dependence of density in molten salts across a range of salt systems, and models appear to be well regularized. While the ideal model accurately predicts the order of magnitude of LiF‐BeF_2_‐ZrF_4_ density, the temperature‐derivative $\frac{\partial \rho }{\partial T}$ (and therefore thermal expansion coefficient *a*_v_) is under‐predicted, resulting in density overprediction above 820 K and underprediction below 820 K. Further, the ideal mixing model overpredicts the density of 53‐24‐23 LiF‐NaF‐BeF_2_ by 75.51 kg m^−3^, the density of 49‐36‐15 LiF‐NaF‐BeF_2_ by 30.77 kg m^−3^, and the density of 49‐15‐36 NaF‐KF‐BeF_2_ by 86.57 kg m^−3^. As such, while ideal models can be used to make predictions even when there is no experimental data, it is clear that the performance of the ideal mixing model is strongly dependent on the system‐specific chemical interactions, which cannot be known a priori. While the RK expansions generally improve the prediction compared to ideal mixing, the improvements are not systematic. For example, RK‐predicted $\left|\frac{\partial \rho }{\partial T}\right|$ can differ significantly from experiments (+45% for NaF‐LiF‐BeF_2_ and −10% LiF‐BeF_2_‐ZrF_4_) as shown in Figure 3a. Meanwhile, for LiF‐NaF‐UF_4,_ density is significantly underpredicted (Δ*ρ* ∼ 200 kg m^−3^) for 65‐15‐20 LiF‐NaF‐UF_4_, but overpredicted (Δ*ρ* ∼ 180 kg m^−3^) for 46‐50‐4 LiF‐NaF‐UF_4_ using RK ternary coefficients in Figure 3b. As such, the magnitude and direction of errors are highly dependent on system, composition, and temperature, and are difficult to predict.

**Figure 4:**
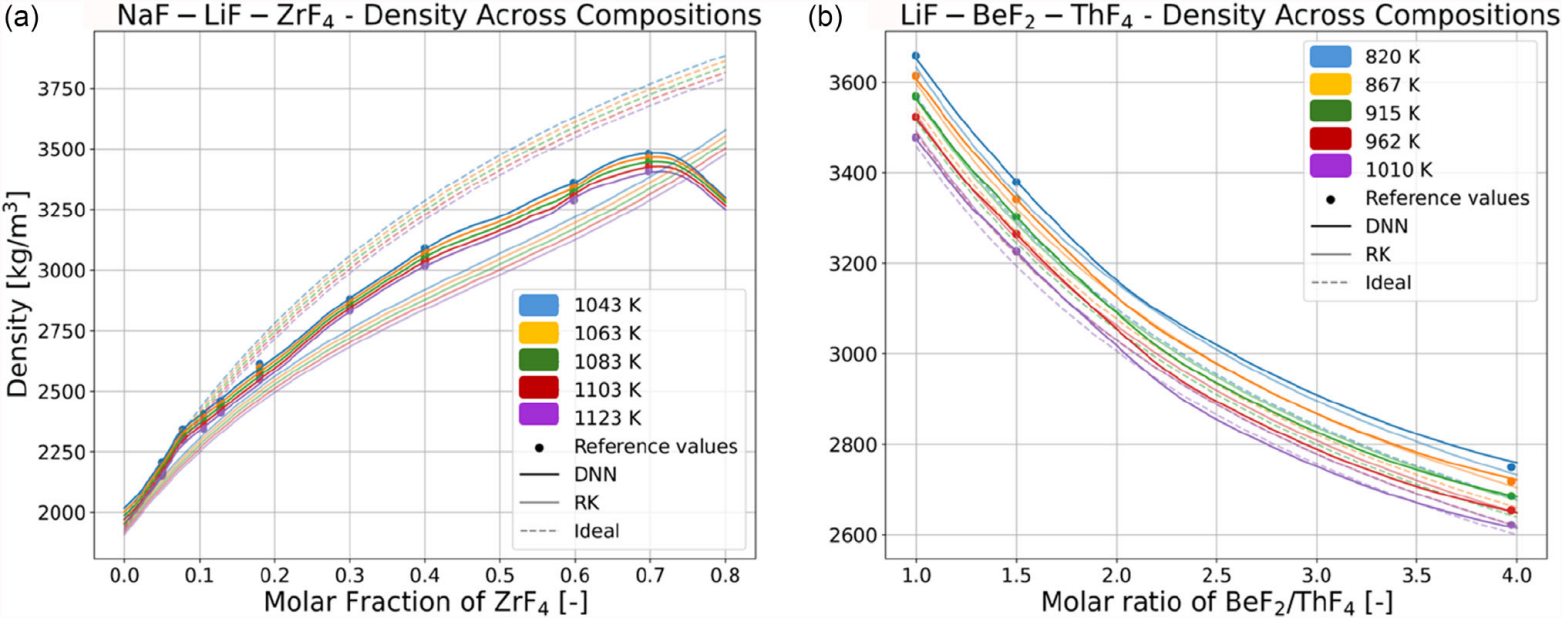
Reference values (points) and predictions of molten salt density by DNN (solid lines), RK (semi‐transparent lines), and ideal mixing (dashed semi‐transparent lines) models at different temperatures of a) NaF‐LiF‐ZrF_4_ across molar fraction of ZrF_4_, with NaF and LiF in a fixed 2:3 molar ratio b) LiF‐BeF_2_‐ThF_4_ across the molar fraction ratio between BeF_2_ and ThF_4_, with LiF fixed at a constant 70% mol. The sampling of the MSTDB‐TP and predictions of all models are made at temperatures of 1043, 1063, 1083, 1103, and 1123 K for the system depicted in a), and at temperatures of 820, 867, 915, 962, and 1010 K for the system depicted in b). In all cases, RK expansions include parameters modeling up to pseudobinary material interactions.

Meanwhile, the DNN accurately interpolates density across temperature. The accuracy of the model when interpolating across composition is more important to ascertain, given the scarcity of data and impossibility of sampling across molar fraction which has led to the implementation of the TL workflow. To showcase the accuracy of the DNN model across composition when compared to the alternative models, density predictions across varying compositions of NaF‐LiF‐ZrF_4_ and LiF‐BeF_4_‐ThF_4_ (challenging systems identified in Ref. [20]) are shown in **Figure** **4**. Figure 4a shows the density predicted for the former system across the molar fraction of ZrF_4,_ and Figure 4b shows predictions for the latter system across the ratio between molar fractions of BeF_2_ and ThF_4_. The MAE and MAPE are calculated for all prediction models across the available density data and shown in **Table** **4**.

**Table 4:** MAE and MAPE calculated for the predictions made by the DNN with TL, the binary RK expansion and ideal mixing (ideal) on the systems shown in Figure 4.

	Overall		
	DNN	RK	Ideal
MAE $\left[\frac{\mathrm{kg}}{m^{3}}\right]$	5.056	87.27	107.3
MAPE [%]	0.192%	3.142%	3.537%

As shown in Figure 4, the density increases with a higher proportion of components with relatively higher molar weight (ZrF_4_ in the NaF‐LiF‐ZrF_4_ and ThF_4_ in LiF‐BeF_2_‐ThF_4_) as expected. The DNN predicts with a MAE of 8.831 kg m^−3^ and a MAPE of 0.332%, a significantly lower error than that attained by RK (MAE of 87.27 kg m^−3^, MAPE of 3.142%) and ideal mixing (MAE of 107.3 kg m^−3^, MAPE of 3.537%). This is particularly salient in the case of NaF‐LiF‐ZrF_4_, where the ideal and RK models diverge from the experimental data towards higher mole fractions of ZrF_4_ as shown in Figure 4. Ideal mixing overpredicts density in the NaF‐LiF‐ZrF_4_ system and underpredicts it in the LiF‐BeF_2_‐ThF_4_, whereas RK expansions do the inverse. The DNN does not appear to either systematically under‐ or over‐predict density, although there is a fall in the curves at the highest concentrations of ZrF_4_, where there is no MSTDB‐TP data available. Thus, these results show the DNN interpolates density across composition more accurately than ideal mixing and RK expansions.

TL‐trained DNNs have been shown to predict density across composition and temperature more accurately than ideal mixing and RK expansions. To showcase the increased accuracy and generalizability achieved through the TL process, the predictions of models trained through this process are compared with those of DNNs trained directly on MSTDB‐TP data. To do this, four additional 80%/20% training/test set splits are created using the MSTDB‐TP with different seed values. The new training sets are used to train four additional DNNs through the TL process previously described, for a total of five TL‐trained DNNs (the first one corresponding to the DNN whose predictions have already been showcased). Furthermore, five DNNs consisting of four hidden layers of 128 neurons each are trained directly on the five training sets generated using the MSTDB‐TP data across 10 000 epochs, using a learning rate of *η *= 5E‐4 and a batch size of 400, with the loss function chosen being the MSE. The validation loss calculated on 20% of the training set is evaluated at each ten epochs, and the model that predicts with the lowest validation loss is preserved. All DNNs are evaluated on their corresponding test sets, the standard metrics are calculated on those predictions, and the results are shown in **Table** **5**.

**Table 5:** MAE, MAPE, and coefficient of determination (r^2^) were calculated for the predictions made by five DNNs trained through the TL process using different splits of the MSTDB‐TP dataset, and by five DNNs trained directly on the same splits of the MSTDB‐TP dataset. Different models whose results are displayed on the same column were trained and evaluated on the same training/test set split.

	Directly trained neural network				
	1st Model	2nd Model	3rd Model	4th Model	5th Model
MAE $\left[\frac{\mathrm{kg}}{m^{3}}\right]$	9.899	6.093	9.270	47.04	50.01
MAPE [%]	0.401	0.248	0.385	1.818	1.978
r^2^ [−]	0.9996	0.9998	0.9991	0.9887	0.9917
Av. MAE $\left[\frac{\mathrm{kg}}{m^{3}}\right]$	24.46				

	Neural network trained through TL				
	1st Model	2nd Model	3rd Model	4th Model	5th Model
MAE $\left[\frac{\mathrm{kg}}{m^{3}}\right]$	6.739	5.598	16.06	5.335	9.298
MAPE [%]	0.265	0.237	0.633	0.201	0.357
r^2^ [−]	0.9993	0.9995	0.9971	0.9996	0.9994
Av. MAE $\left[\frac{\mathrm{kg}}{m^{3}}\right]$	8.606				

The models trained through the TL process predict density with an MAE of 8.606 kg m^−3^ on average, whereas the directly trained DNNs predict density with an average MAE of 24.46 kg m^−3^. For all but one model, the MAE and MAPE of the DNNs trained through TL are lower than for the DNNs trained directly on MSTDB‐TP data. These results show that training DNNs through the TL process is not a guarantee of obtaining a model that predicts density with a higher accuracy than one trained directly on MSTDB‐TP data, but it is effective in generating models that are more accurate on average, regularizing models to not reach the high errors that can be seen for the 4th and 5th directly trained models.

**Figure 5:**
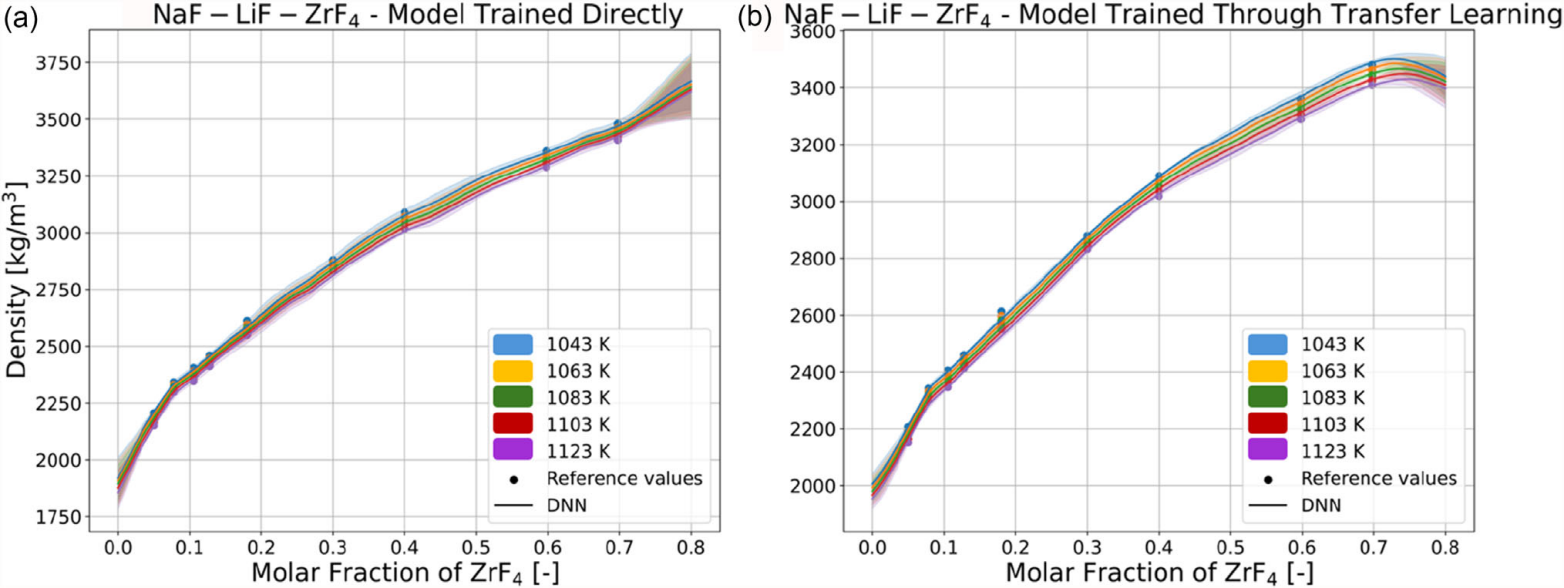
Reference values (points) and predictions of molten salt density (solid lines) of the NaF‐LiF‐ZrF_4_ system across molar fraction of ZrF_4_, made by ensembles of a) five DNNs trained directly on MSTDB‐TP data, and of b) five DNNs trained through the TL process. The predictions for both ensembles are shown with confidence interval bands of 95% colored in the same color corresponding to the temperature shown for the temperatures in the legend.

**Figure 6:**
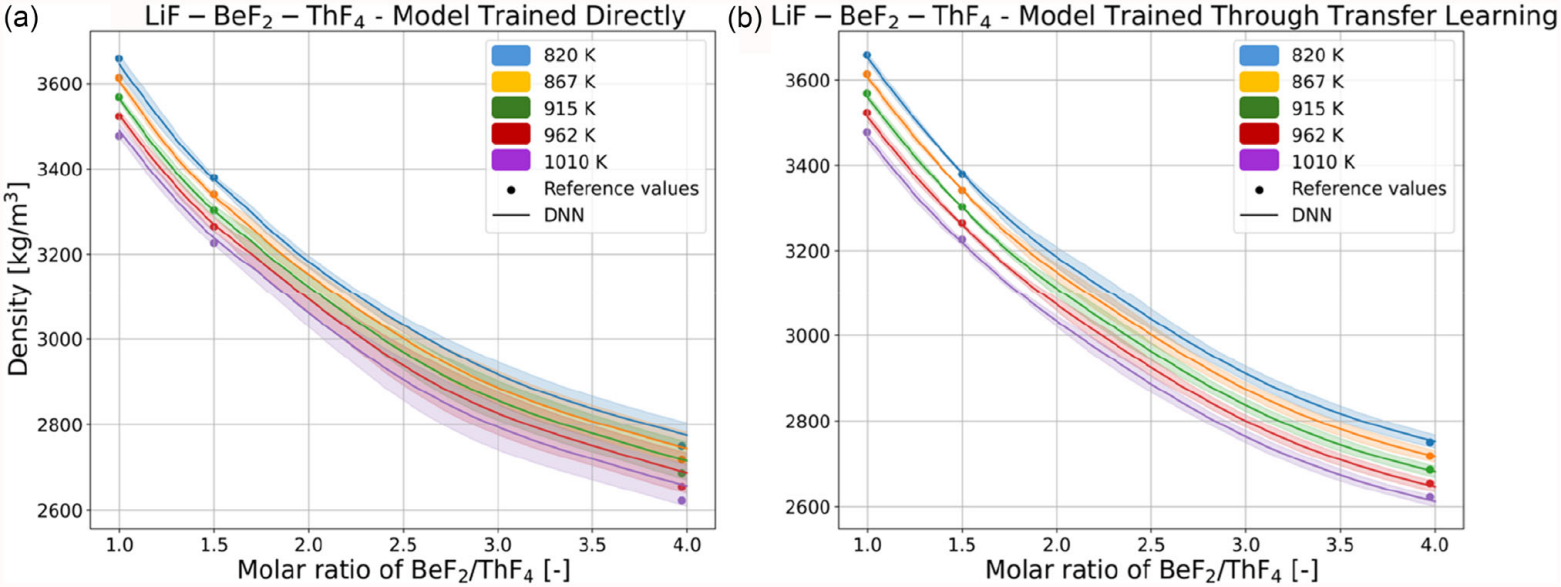
Reference values (points) and predictions of molten salt density (solid lines) of the LiF‐BeF_2_‐ThF_4_ system across the ratio of molar fractions of BeF_2_/ThF_4_, made by ensembles of a) five DNNs trained directly on MSTDB‐TP data, and of b) five DNNs trained through the TL process. The predictions for both ensembles are shown with confidence interval bands of 95% colored in the same color corresponding to the temperature shown for the temperatures in the legend.

The effectiveness of the TL process is further showcased by qualitatively comparing the behavior of DNNs trained directly and through the TL process in data‐sparse regions. **Figure** **5** shows the predictions of the same system shown in Figure 4a as made by ensembles of the five models trained directly on MSTDB‐TP data and the five models trained through TL, whereas **Figure** **6** shows the predictions of the system shown in Figure 4b made by the same ensembles. The predictions of the individual models are aggregated by averaging, and the confidence intervals of the predictions of each ensemble is calculated using Equation (6).^[^ ^50^ ^]^ The *z* value used in Equation (6) is 1.96, corresponding to a 95% confidence interval. The number of measurements “*n*” is 5 since a prediction is made by every model of each ensemble, and σ represents the standard deviation between the predictions.

(6) $$ci=\frac{z\sigma }{\sqrt{n}}$$

For both Figure 5 and 6, the predictions of the ensemble of directly trained models are associated with a higher level of uncertainty than for the TL‐trained ensemble, which is evidenced by the wider uncertainty bands, and which is indicative of the models being less stable, their predictions oscillating more widely around the reference values. Concerning Figure 5, Figure 4 showed how DNN predictions diverged at the highest concentrations of ZrF_4,_ where there is no MSTDB‐TP data available, and this is reflected in the widening uncertainty bands at the higher ZrF_4_ concentrations for both ensembles, evidencing how the instability of predictions when extrapolating across compositions is not exclusive to the TL‐trained models. The effectiveness of the TL approach is especially notable in Figure 6 when comparing the predictions of the directly trained ensemble, which are inaccurate and whose uncertainty bands overlap heavily at the highest BeF_2_/ThF_4_ ratios, with those of the TL‐trained ensemble, more accurate and whose uncertainty bands are almost completely separate. From the results seen in Table 4, Figure 5, and 6, we can conclude that the TL process is effective in training DNNs to be more accurate and better behaved than DNNs trained directly on sparse data. The RK data effectively regularizes DNNs to predict density as one would expect density curves to behave in reality, and more stably in regions with a dearth of available data.

In examples presented in Figure 3 and 4, and the overall dataset presented in Figure S1, Supporting Information, we have shown significant challenges in modeling fluoride salts using existing modeling methods. While the density of pure symmetric salts (e.g., alkali metal halides) can be modeled via the additive molar volume of their constituents,^[^ ^51^, ^52^ ^]^ relationships have not been established for non‐symmetric salts, and even less so for salt mixtures that exhibit strong non‐idealities that affect density in ways that cannot be systematically predicted. While empirical correlations based on ionic radii, charge, and quasichemical models have been used for modeling salt density,^[^ ^51^, ^53^, ^54^ ^]^ such approaches still rely on system‐specific fitted parameters, and universal correlations cannot be uncovered. Here, DNNs are used to learn the underlying relationships between chemical‐structural features of salt constituents and their resultant mixture density across 448 pure, pseudobinary, and pseudo‐ternary fluoride and chloride salt systems. Across the dataset of chlorides and fluorides (Section S6, Figure S2 and Figure S3 in Supporting Information), we have demonstrated that this method is robust (although section S4 of the Supporting Information shows that ideally more data should be included in future work to improve the predictions of the model on data points that are significantly outside of the training distribution), and that density, a key thermodynamic property, can be successfully correlated to underlying atomic properties across the periodic table. This presents promising opportunities for developing new theories and significantly improving our fundamental understanding of molten salt properties.

## 3 Conclusion

In summary, a chemistry‐informed approach for training more generalizable DNNs was developed and shown to accurately predict density in molten salts across a vast composition‐temperature space for the first time. The model predicts the density of salts in the MSTDB‐TP with an overall MAPE of 0.265%, a higher accuracy than that achieved by RK on pseudobinary systems (0.768%) and RK on pseudo‐ternary systems (1.711%). The error achieved by the DNN is within experimental uncertainty, demonstrating accuracy that might be limited only by the inaccuracies in the experimental methods used to acquire training data. Using the DNN on data‐sparse molten salt systems shows it can smoothly and accurately predict density at a high resolution of temperature and compositional ratios significantly better than state‐of‐the‐art semi‐empirical models, which require large datasets of subcomponent mixtures, and are limited by functional form for capturing complex interactions. While the semi‐empirical models are still necessary to generate data for the TL process, the DNN can predict density for systems for which subcomponent data is not available. The DNN also predicts density with a higher accuracy than the ideal models, which do not require as much subcomponent information but perform significantly worse due to an inability to account for significant contributions from excess density. These qualities make the DNN model suitable for interpolating the available data for density in molten salts, indicating that this approach can be extended for the prediction of other critical molten salt properties for which information is available, such as viscosity and heat capacity. Furthermore, the developed method can be applied to studying a wide range of materials mixtures including mixed oxides for thermal energy storage and energy conversion systems, nitrate molten salts used in hydrocarbon mixtures, thermal energy storage, and many more clean energy applications, although further work must be done to assess the applicability of the model towards these salts and to other materials, and to expand this applicability by incorporating a wider variety of data from more sources.^[^ ^55^, ^56^, ^57^, ^58^, ^59^ ^]^

## Conflict of Interest

The authors declare no conflict of interest.

## Supporting information

Supplementary Material

## Data Availability

Both the code and the data used to generate the models can be found in the following GitHub repository: https://github.com/JulianBarra/Transfer‐Learning‐DNN‐for‐Molten‐Salts.
