# Supplementary material for: Toward a Generalizable Prediction Model of Molten Salt Mixture Density with Chemistry‐Informed Transfer Learning
Source: Chemphyschem. 2025 Oct 14;26(23):e202500273. doi: 10.1002/cphc.202500273 (PMC12677718; doi:10.1002/cphc.202500273)
Supplement: Supplementary file 1 — Supplementary Material [file CPHC-26-e202500273-s001.pdf]

Supporting Information

Julian Barra[1], Shayan Shahbazi[2], Anthony Birri[3], Rajni Chahal[3], Ibrahim Isah[1], Muhammad Nouman Anwar[1], Tyler Starkus[2], Prasanna Balaprakash[3], Stephen Lam[1*]*

[1]Department of Chemical Engineering, University of Massachusetts Lowell, Lowell, MA-01854, USA

[2]Argonne National Laboratory, Lemont, IL-60439, USA

[3]Oak Ridge National Laboratory, Oak Ridge, TN-37830, USA

*Stephen Lam (Stephen_Lam@uml.edu)

S1. Downselection process for JARVIS-CFID descriptors.

The chemical information of the salts is encoded by adding descriptors corresponding to material properties obtained from the JARVIS-Tools Classical Force Field Inspired Descriptors (JARVIS-CFID)[1] database of ab-initio calculations. JARVIS-CFID contains 1557 chemical-structural-charge descriptors for any formula (e.g., Pb, LiF, CH_4) at 0K. These descriptors were not systematically tested in order to be reduced (although the effect of the final list of descriptors in improving the predictions of the model when compared to models trained only on the atomic numbers of the elements in the salts can be found section SP), rather, the discarded descriptors were chosen according to some prior considerations. Of the 1557 descriptors, 438 correspond to property values calculated for the elements in each compound averaged over all of their atoms, including chemical, structural and charge properties, but for the individual elements in the compounds, this includes descriptors calculated for when these elements are at an equilibrated 0 K structure.

The rest of the descriptors correspond to certain structural properties for a particular Python object describing the positions of ions in a unit cell structure. We do not posses the structures for the molten salts whose information is registered in the MSTDB-TP, obtaining those structures for all data points in the MSTDB-TP and the RK dataset through the use of molecular dynamics is beyond the scope of this work, and the most similar available structures correspond to the optimized crystal structures of the individual compounds in the mixture at 0 K. Molten salts being in the liquid state, this structure is not preserved. The looser structure of the salts in the liquid state leads us to believe the aforementioned 438 descriptors calculated and averaged at the ionic level are better suited at capturing the ionic interactions from which density emerges.

Some of these 438 descriptors were discarded due to corresponding to the results of operations between the other descriptors listed in the supporting information (i. e., one of the descriptors corresponds to the averaged fusion heat of the compound elements divided by the averaged molar volume of the elements). These descriptors were considered a proiri redundant for training a deep neural network, and are therefore discarded, with the descriptors representing pure material properties being used instead. There is still ongoing work on the more systematic reduction of descriptors, but it is considered out of scope for this work, whose primary objective is to put forward the proof of concept for a methodology to train DNNs for increased transferability and generalizability when predicting for a material with very little data available, and for which very few attempts for predicting using ML algorithms have been made.

The final list of descriptors included from JARVIS-CFID can be found in section S2.[2]

S2. Properties included in molten salt datasets.

Material descriptors used to encode information about the molten salts were obtained as properties calculated and available for materials in the JARVIS-CFID properties database. The full list of descriptors obtained for every one of the salt compounds in the mixture is as follows[2]:

- Energy per atom of an element from JARVIS-DFT.
- Elemental shear modulus from JARVIS-DFT.
- Elemental bulk modulus from JARVIS-DFT.
- OptB88vdW bandgap during SCF for an element
- OptB88vdW bandgap during linear optics for an element.
- Voronoi coordination number of an element-crystal structure.
- Number of unfilled d-orbitals.
- Number of valence d-orbitals.
- Number of unfilled s-orbitals.
- Number of valence s-orbitals.
- Number of unfilled p-orbitals.
- Number of valence p-orbitals.
- Number of unfilled f-orbitals.

- Number of valence f-orbitals.
- First ionization energy of an element.
- OQMD bandgap of an element.
- Electron affinity.
- Volume per atom of an element.
- Heat of fusion of an element.
- OQMD energy per atom.
- Polarizability.
- Atomic number.
- Electronegativity.
- Row number in the periodic table.
- Column number in the periodic table.
- Atomic mass.
- Atomic radius.
- Thermal conductivity.
- Molar volume.

- Boiling point.
- Melting point.
- Average ionic radius.

JARVIS-CFID descriptors can be calculated for elements and they can be calculated for compounds, but the latter case, what is actually calculated is the values for the descriptors of each one of the elements in the compounds, and these values are averaged for each of the elements in the formula. For example, the value of a descriptor "Y_{CH4}" for methane (CH_4) is calculated from the values of the same descriptor for carbon "Y_C" and for hydrogen "Y_H" according to equation S1.

$$Y_{CH4} = \frac{(1 \cdot Y_C + 4 \cdot Y_H)}{5} \tag{S1}$$

Other descriptors obtained for each compound in the mixture are:

- Molar fraction of the compound.
- Molar mass of the compound.

With the first one being obtained from the dataset itself and the second one being calculated for each compound using the Molmass library. Lastly, there are two descriptors which are not calculated for each compound, rather they being only one for the entire salt:

- Weighted molar mass.
- Sampling temperature.

The first one corresponds to the molar weights of the compounds multiplied by the molar fraction of each in the mixture, and the second one is the temperature used to generate the data point. With these last two descriptors being the only exception, each one of the material properties shown in the list is obtained from Jarvis for each one of the four compounds present in the mixture. The descriptors are concatenated to create a vector of properties, and those vectors are in turn concatenated in the order the compounds are represented. Mixtures of sub-pseudoquaternary order are zero-padded so data points for mixtures of all possible orders still have the same number of columns (i.e., for a mixture $[\mathbf{C_1}, \mathbf{C_2}, \mathbf{0}, \mathbf{0}]$, the vector of descriptors for the forst compound are added ($\mathbf{MW_1}, \mathbf{X_1}, \mathbf{A_1}, ...$), then the ones for the second compound ($\mathbf{MW_2}, \mathbf{X_2}, \mathbf{A_2}, ...$), and the rest of the descriptors, save for the temperature and the molecular weight, are left as 0. Multiplying the 32 Jarvis descriptors for four possible compounds means the total number of input dimensions obtained from Jarvis is 128. To those 128 inputs, the descriptors for the molar fraction and molar masses are added for each compound, adding up to 136. The last two descriptors correspond to the temperature and the molecular weight, and are added at the end once, representing the whole mixture. All of these descriptors add up to a total of 138 inputs. To give an example, if one performs this process to generate the descriptors associated with the first three JARVIS-CFID descriptors, one obtains the following ones:

- Energy per atom of an element from JARVIS-DFT for compound in position 1.
- Energy per atom of an element from JARVIS-DFT for compound in position 2.
- Energy per atom of an element from JARVIS-DFT for compound in position 3.
- Energy per atom of an element from JARVIS-DFT for compound in position 4.

- Elemental shear modulus from JARVIS-DFT for compound in position 1.
- Elemental shear modulus from JARVIS-DFT for compound in position 2.
- Elemental shear modulus from JARVIS-DFT for compound in position 3.
- Elemental shear modulus from JARVIS-DFT for compound in position 4.
- Elemental bulk modulus from JARVIS-DFT for compound in position 1.
- Elemental bulk modulus from JARVIS-DFT for compound in position 2.
- Elemental bulk modulus from JARVIS-DFT for compound in position 3.
- Elemental bulk modulus from JARVIS-DFT for compound in position 4.

This is repeated for all descriptors with the exception of the last two, the weighted molar mass and the sampling temperature.

S3. Evaluation of the model trained only on atomic number descriptors.

To showcase the effectiveness of adding the descriptors to featurize the dataset, we use the new training and test created to test the transferability of the DNN model through the process described in S4. The datasets are featurized two times, the first time using only the atomic number descriptor available in JARVIS (which corresponds to the atomic numbers of all elements in each compound averaged across all atoms), the sampling temperature, and the molar fractions of the compounds in the mixture, and the second time using all 138 descriptors shown previously in S1. Two different models are trained using the transfer learning methodology outlined in the manuscript, one of them on the first dataset and the other one trained on the fully featurized dataset. The metrics for the predictions of the first model on the test set have been calculated and can be seen in Table S1.

Table S1. Mean absolute error (MAE), mean absolute percentage error (MAPE), and coefficient of determination (r^2) calculated for the predictions made on a test set by a deep neural network (DNN) trained through the transfer learning process on a training set featurized using only atomic number descriptors.

	**DNN**
**MAE** $\left[\frac{kg}{m^3}\right]$	117.8
**MAPE** [%]	5.038 %
$\mathbf{r^2}$ [−]	0.8916

For comparison, the metrics for the predictions on the test set made by the DNN trained through the transfer learning process on a training set featurized using all 138 descriptors can be seen in Table S2

Table S2. Mean absolute error (MAE), mean absolute percentage error (MAPE), and coefficient of determination (r^2) calculated for the predictions made on a test set by a deep neural network (DNN) trained through the transfer learning process on a training set featurized using all 138 descriptors.

	**DNN**
**MAE** $\left[\frac{kg}{m^3}\right]$	80.16
**MAPE** [%]	3.259%
$\mathbf{r^2}$ [−]	0.9672

The results of using only the atomic number descriptors compare unfavorably to those of using the Jarvis descriptors used in this work. While there is further work to be done in the systematic reduction of descriptors, these results show the descriptors are improving the performance of the models.

S4. Evaluation of DNN models on a test set generated to have completely different compositions

Earlier iterations of this work added the permutations of all molten salt compositions before performing the 80%/20% training/test set split and before sampling across temperature in order to generate density data points. Several concerns about the possibility of data leakage were raised due to the possibility of several permutations of the same composition at the same temperature appearing in both the training and test sets simultaneously. This led to the procedure described in the main body of the manuscript, in which all molten salt compositions of the MSTDB-TP are sampled across temperature first, the data is split in a 80%/20% ratio second, and then all possible permutations are added to the training and test sets to ensure that no permutations of any data point found in the training set can also be found in the test set.

A more extreme strategy for preventing data leakage is to perform the training/test set split before sampling these compositions across temperature and adding any permutations. Doing this ensures that the compositions that are present in the training set cannot be found in the test set at all, therefore testing the limits of the transferability and generalizability of the model. Due to the small size of the MSTDB-TP, this leads to a very high probability of compositions with elements present in one set and not at all in the other. The results of doing this can be seen in Table S3.

Table S3. Mean absolute error (MAE), mean absolute percentage error (MAPE), and coefficient of determination (r^2) calculated for the predictions made by a deep neural network (DNN) trained through the transfer learning process on a training set possessing completely different compositions than those present in the test set.

	**DNN**
**MAE** $\left[\frac{kg}{m^3}\right]$	80.16
**MAPE** [%]	3.259%
$\mathbf{r^2}$ [−]	0.9672

These results are not included in the main body because is not considered adequate to compare them with the results of using the RK expansions shown in Figure 2 of the main text of the article: RK models of the highest possible order, like those shown in the figure, are specifically fit to the compositions they are making predictions for, whereas the DNN is being asked to predict for compositions missing in the training set entirely. The availability of molten salt data in general is low, with some compositions very sparsely populated, and splitting the dataset as the very first step can lead to the test set possibly containing compounds and elements it might not have seen at all during training.

A possible point of comparison could be the predictions made by ideal mixing, which do not require all the information that is needed to fit RK polynomial equations. Table S.Y shows the metrics of the predictions made by the ideal mixing assumptions, showing a MAPE of % 3.388 and an MAE of 93.43 kg/m^3, both of them higher than the MAPE of % 3.259 and the MAE of 80.16 kg/m^3 for the predictions of the DNN model. The ideal mixing assumption does show a r^2 of 0.9765, outperforming the r^2 of 0.9672 of the DNN model, but it bears mentioning that the

predictions made by the ideal mixing assumption shown in section S3 are made on the entire MSTDB-TP dataset, which includes pure salts. In these cases, the ideal mixing assumption, which uses the densities of the compounds in the mixture, is “predicting itself”, therefore biasing the metrics in favor of ideal mixing. Ideal mixing also has the advantage that density data of the subcomponents must be available for it to make predictions for mixtures of a higher order, whereas it is possible for the test set generated for this test to contain compounds and elements not present at all during training, a type of extrapolation which is very hard for DNNs to perform. We feel confident in saying that the models would therefore be a better alternative than ideal mixing when making predictions for unknown molten salt mixtures.

S5. Overprediction of molten salt density using ideal mixing approximation

Figures 3 and 4 in the main body show the ideal mixing rule tends to predict density above their reference values. Further analyzing ideal mixing predictions shows this to be a generalized tendency across the dataset. Figure S1 shows a parity plot for the predictions of density in pseudo-binary and pseudo-ternary molten salt mixtures and a histogram for the predictions with respect to reference. The metrics for the predictions are calculated and can be seen in Table S4.

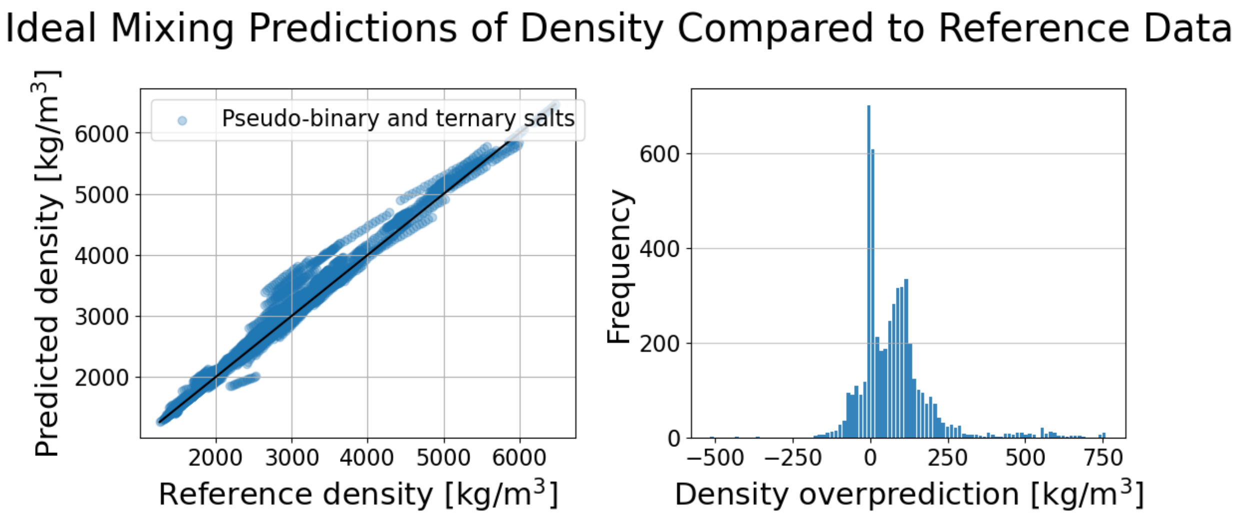

Figure S1. Parity plot for the predictions of density by the ideal mixing rule for the pseudo-binary and pseudo-ternary salts mixtures in the MSTDB-TP (left) and histogram for the deviations of mixture density predictions by the ideal mixing rule when compared to the reference MSTDB-TP density data (right).

Table S4. Mean absolute error (MAE), mean absolute percentage error (MAPE), and coefficient of determination (r^2) calculated for the predictions made by ideal mixing for the salts of the MSTDB-TP dataset.

	**DNN**
**MAE** $\left[\frac{kg}{m^3}\right]$	93.43
**MAPE** [%]	3.388%
$\mathbf{r^2}$ [−]	0.9765

Ideal mixing overpredicts mixture density by an average of 76.6 kg/m^3. Possible explanations for this behavior have been given in the main body of the text, but a specific physical mechanism for this has not been outlined in the molten salt literature to our knowledge. The existence of such behavior is the driving factor behind the application of RK expansions to adjust for the excess density.

S6. Performance of Deep Neural Network Model Across MSTDB

Figure S2 and S3 show (a) the mean absolute errors (MAE) of DNN predictions and (b) the number of data points that were used to train the DNN model. The plots show the MAE for systems containing specific chloride compounds and fluoride compounds, respectively. As shown in Figures S2 and S3, most systems exhibit low MAE of < 10 kg/m^3 (<1%) within experimental uncertainty even for cases where a relatively small amount of data was used in training. This demonstrates the overall model generality across the MSTDB and ability to learn relationships across the periodic table based on fundamental chemical-structural features. Prediction across fluoride salts (Figure S2) are highly accurate across the board, with experimental data more densely populated relative to chloride salts (Figure S3). The $PuCl_3$-NaCl pseudo-binary and pure $ZrCl_4$ systems (Figure S3) are outliers exhibiting a relatively high error of 185 kg/m^3 and 205 kg/m^3 (~6%), respectively. Data from $ZrCl_4$ originated from a study in 1965 with a 1% uncertainty, and only data for pure $ZrCl_4$ is available[3]. Meanwhile, data on NaCl-$PuCl_3$[4] reported an uncertainty of 1.5%. Here, it is noteworthy that the chemical complexity of Plutonium (multiplicity of possible oxidation states, phases, and chemical complexes formed during synthesis), and lack of data on other systems (pseudo-binary, ternary or quaternary) containing $PuCl_3$, could have contributed to higher model error.

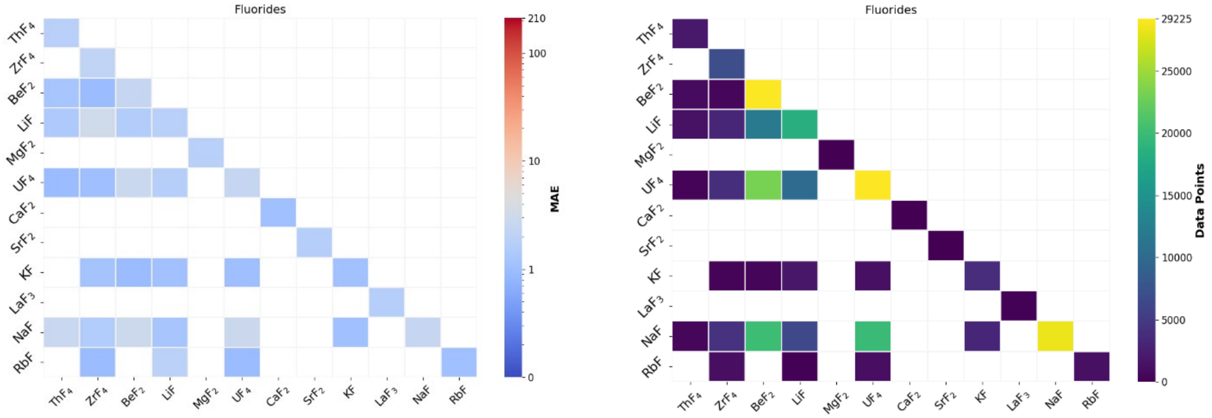

a **b**

Figure S2: a) Mean absolute error of DNN predictions for fluoride systems in the MSTDB containing a given fluorine compound or set of compounds, b) the corresponding number of datapoints used in training.

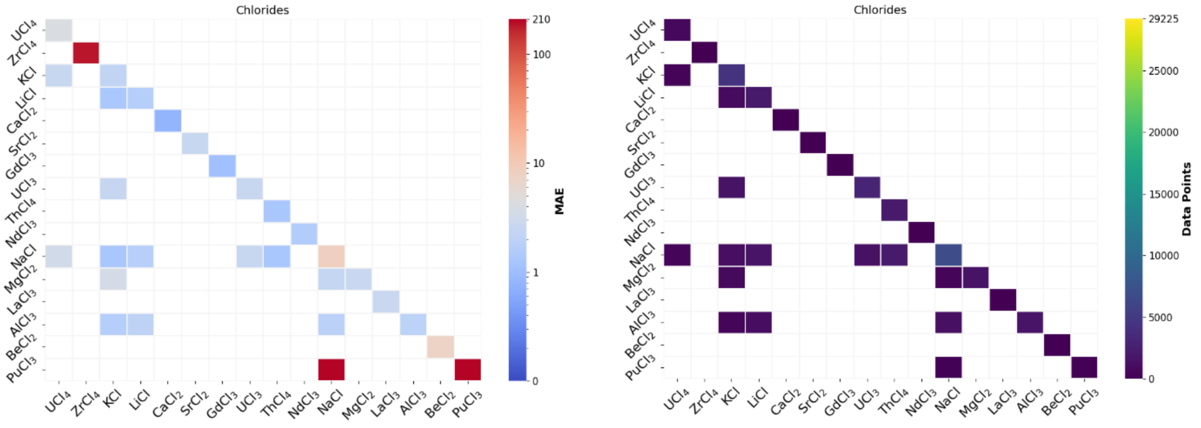

a **b**

Figure S3: a) Mean absolute error of DNN predictions for chloride systems in the MSTDB containing a given chlorine compound or set of compounds, b) the corresponding number of datapoints used in training.

References

1. Choudhary K, Garrity KF, Reid ACE, DeCost B, Biacchi AJ, Hight Walker AR, et al. The joint automated repository for various integrated simulations (JARVIS) for data-driven materials design. NPJ Comput Mater. 2020 Dec 1;6(1).

2. Choudhary K, Decost B, Tavazza F. Machine learning with force-field-inspired descriptors for materials: Fast screening and mapping energy landscape. Phys Rev Mater. 2018 Aug 3;2(8).

3. Nisel'son LA, Stolyarov VI, Sokolova TD. Some Properties of Liquid Zirconium Tetrachloride. Russian Journal of Physical Chemistry. 1965;39:1614.

4. Karlsson TY, Middlemas SC, Nguyen MT, Woods ME, Tolman KR, Glezakou VA, et al. Synthesis and thermophysical property determination of NaCl-PuCl3 salts. J Mol Liq. 2023 Oct 1;387.
